# Synthesis and Structure-Activity Relationships of Amino Acid Conjugates of Cholanic Acid as Antagonists of the EphA2 Receptor

**DOI:** 10.3390/molecules181013043

**Published:** 2013-10-21

**Authors:** Simonetta Russo, Matteo Incerti, Massimiliano Tognolini, Riccardo Castelli, Daniele Pala, Iftiin Hassan-Mohamed, Carmine Giorgio, Francesca De Franco, Antimo Gioiello, Paola Vicini, Elisabetta Barocelli, Silvia Rivara, Marco Mor, Alessio Lodola

**Affiliations:** 1 Dipartimento di Farmacia, Università degli Studi di Parma, Viale delle Scienze 27/A, Parma I-43124, Italy; 2 TES Pharma S.r.l., Via P. Togliatti, 20, Loc Taverne, Corciano 06073, Italy; 3 Dipartimento di Scienze Farmaceutiche, Università degli Studi di Perugia, Via del Liceo, 1, Perugia 06123, Italy

**Keywords:** cholanic acid, amino acid conjugates, EphA2 antagonists, structure-activity relationships

## Abstract

The Eph–ephrin system plays a critical role in tumor growth and vascular functions during carcinogenesis. We had previously identified cholanic acid as a competitive and reversible EphA2 antagonist able to disrupt EphA2-ephrinA1 interaction and to inhibit EphA2 activation in prostate cancer cells. Herein, we report the synthesis and biological evaluation of a set of cholanic acid derivatives obtained by conjugation of its carboxyl group with a panel of naturally occurring amino acids with the aim to improve EphA2 receptor inhibition. Structure-activity relationships indicate that conjugation of cholanic acid with linear amino acids of small size leads to effective EphA2 antagonists whereas the introduction of aromatic amino acids reduces the potency in displacement studies. The β-alanine derivative **4** was able to disrupt EphA2-ephrinA1 interaction in the micromolar range and to dose-dependently inhibit EphA2 activation on PC3 cells. These findings may help the design of novel EphA2 antagonists active on cancer cell lines.

## 1. Introduction

The erythropoietin-producing hepatocellular carcinoma (Eph) receptors form the largest family of receptor tyrosine kinases (RTKs) and, together with their ephrin ligands, are involved in the processes of cellular communication by virtue of the membrane localization of both Eph receptors and ephrins [1]. To date, fourteen Eph-family members, divided in two classes (EphA and EphB receptors) and eight ephrin ligands have been identified in humans. The bidirectional cell-cell signalling resulting from the Eph-ephrin interaction participates in the modulation of the complex dynamics of cellular migration and adhesion, which are inherently connected to cancer insurgence and progression [2]. Dys-regulation of the Eph-ephrin system activity affects cell-matrix adhesion, cell-cell adhesion, organization of the cytoskeleton and tumor cell survival, leading to increased cellular motility, tumor cell invasion and metastasis [3,4]. High levels of Eph receptors have been found in several cancer types [5] comprising breast, lung, colon, prostate and melanoma, as well as in blood vessels, where they promote tumor angiogenesis [6].

The function of the Eph-ephrin system in tumorigenesis and angiogenesis makes it a promising therapeutic target for the treatment of solid tumors. Several strategies have been applied during the past years to target this system such as monoclonal antibodies, recombinant proteins (*i.e.*, soluble forms of Eph or ephrin) and peptides [7]. An attractive alternative is represented by small molecules able to interfere with the Eph-ephrin system. In this context, a possible approach is represented by heterocyclic compounds able to inhibit the kinase activity of the Eph receptor [8] by occupying its ATP-binding site. However, the lack of selectivity of classical kinase inhibitors remains so far an indisputable limit of this class of compounds [9], the highly conserved sequences of the ATP-binding pocket among protein kinases. A different strategy features the use of small molecules to directly prevent the interaction between the Eph receptor and its ephrin ligand. Few low-molecular weight compounds able to disrupt the Eph-ephrin binding have been recently identified: (*i*) disalicylic acid derivatives that bind the EphA2 and EphA4 receptors with a non competitive mechanism of action [10]; (*ii*) doxazosin, which acts as an agonist on EphA2 and EphA4 receptors [11]; (*iii*) naturally occurring polyphenols and their metabolites, such as gallic acid [12,13].

**Figure 1:**
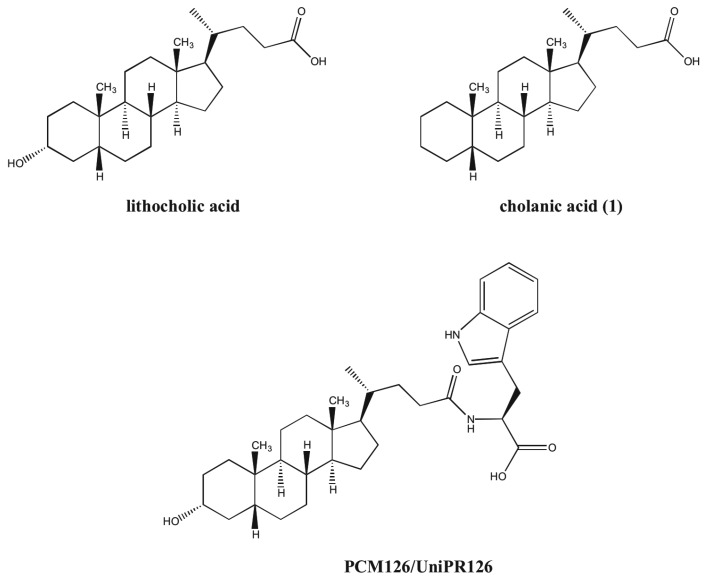
Chemical structure of lithocholic acid, cholanic acid (**1**) and PCM126 (UniPR126).

We have recently described lithocholic acid (LCA, Figure 1) as a competitive and reversible Eph receptor ligand. Functional studies showed that LCA acts as an antagonist of the EphA2 receptor as it inhibits, in a dose-dependent manner, the phosphorylation of EphA2 and blocks PC3 cells retraction and rounding induced by EphA2 stimulation with ephrin-A1 at non-cytotoxic concentrations [14]. Exploration of the structure-activity relationships (SARs) of lithocholic acid derivatives identified cholanic acid (**1**) as a more potent inhibitor of the EphA2-ephrin-A1 interaction (pIC_50_ = 4.91) than LCA (pIC_50_ = 4.24) [15], confirming that the 5β-cholan-24-oic acid structure could serve as an effective scaffold to design improved EphA2 inhibitors. To improve the inhibitory potency and physicochemical properties of lithocholic acid, a series of α-amino acid conjugates of LCA were prepared based on its theoretical binding mode to the EphA2 receptor [16]. As a result of this investigation, the L-Trp conjugate of LCA (known as PCM126/UniPR126) resulted the most potent derivative, able to disrupt the EphA2-ephrin-A1 interaction with a pIC_50_ of 5.69 (Figure 1). Conjugation of LCA with L-Trp not only provided an effective antagonist at the EphA2 receptor but it allowed facing the issue of selectivity *versus versus* known targets of bile acids. Specifically, while LCA is a rather potent agonist of both FXR and TGR5 receptors [17,18] PCM126/UniPR126 does not interact with FXR while it is endowed with activity on TGR5 receptor in the micromolar range (data from an unpublished study).

All together, these findings prompted us to prepare and characterize some α and β-amino acid conjugates of the reference EphA2 antagonist cholanic acid. The newly synthetized compounds were tested on the EphA2 receptor and the gathered SAR data were rationalized through molecular docking simulations.

## 2. Results and Discussion

### 2.1. Chemistry

**Scheme 1:**
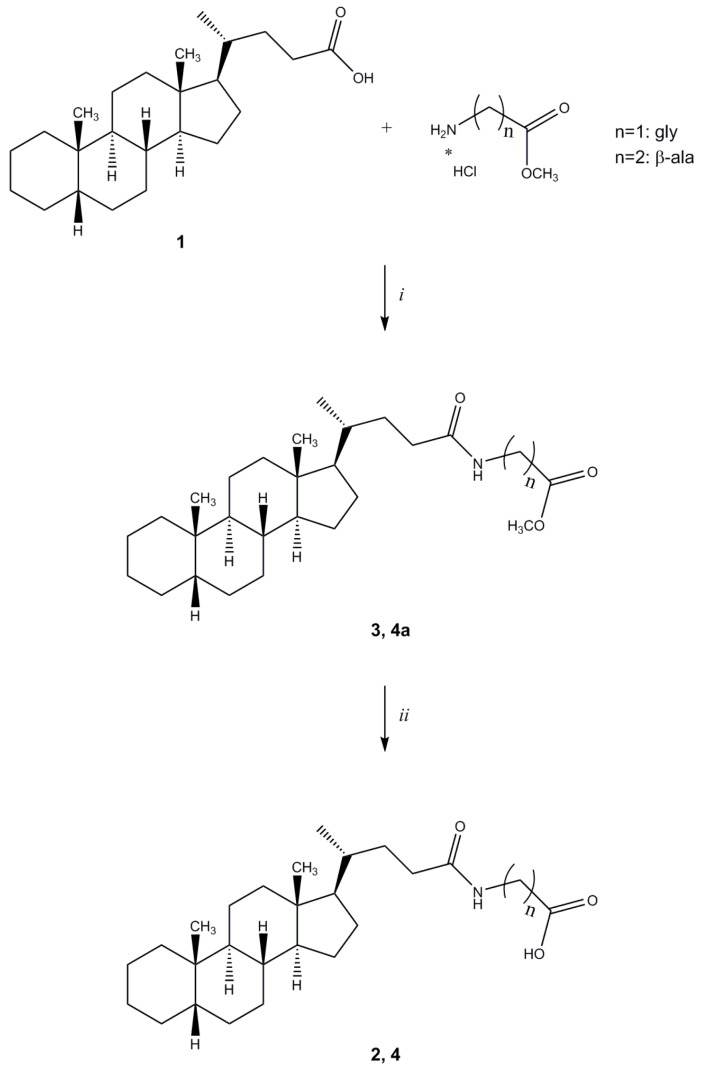
General synthesis of compound **2** and **4**. *^a^*

**Scheme 2:**
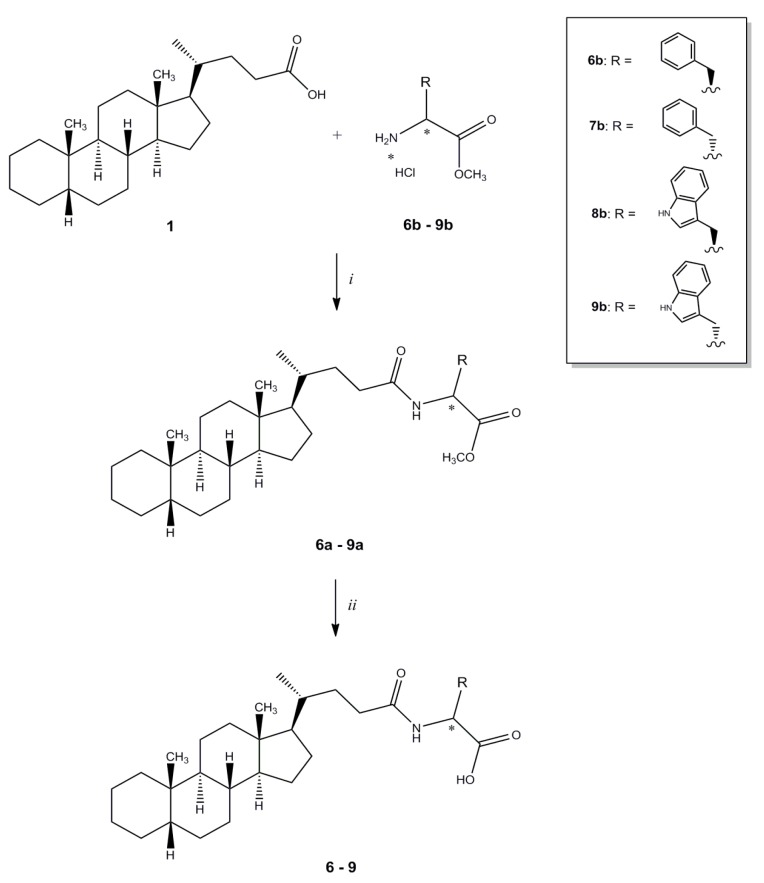
General synthesis of compound **6**–**9**. *^a^*

Cholanic acid (**1**) and methyl ester hydrochlorides of amino acids are commercially available, while compounds **2**–**4**, **6**–**9** were synthesized according to known procedures or minor modifications to those described in ref. [16] (Scheme 1 and Scheme 2). The methyl ester hydrochloride of the appropriate amino acid was reacted with **1**, using *N*-(3-dimethylaminopropyl)-*N'*-ethylcarbodiimide hydrochloride (EDCI) as coupling agent. The ester group of amides **3**, **4a**, **6a**–**9a** was hydrolyzed with NaOH to give compounds **2**, **4**, **6**–**9**.

**Scheme 3:**
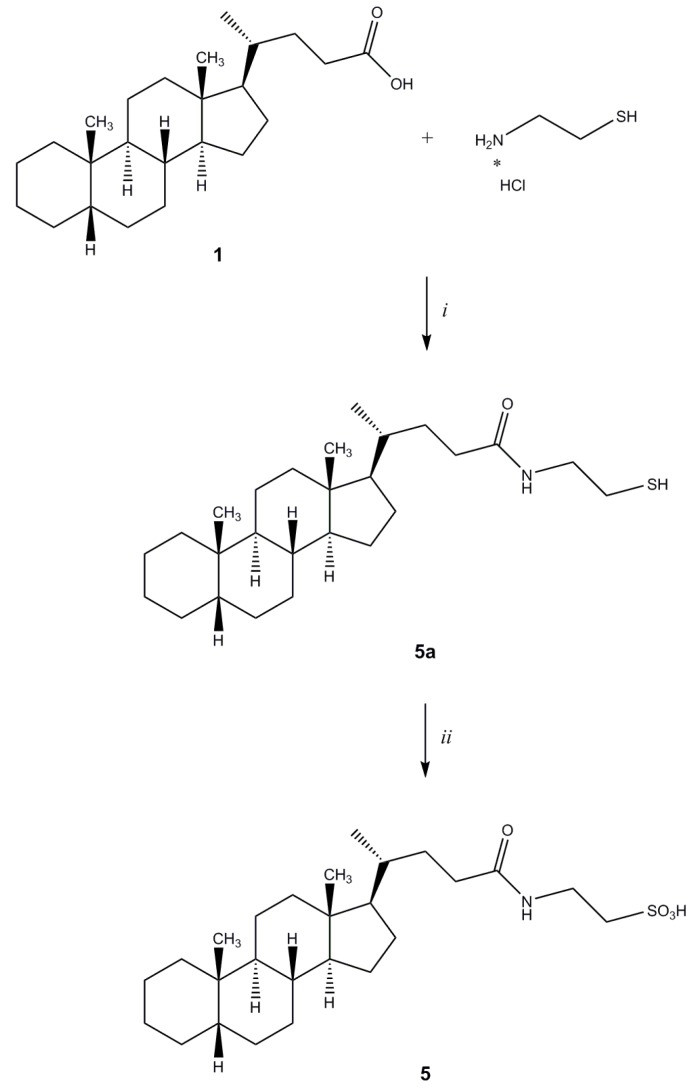
General synthesis of compound **5**. *^a^*

Compound **5** was synthesized according to the procedure reported in Scheme 3. Commercially available cysteamine hydrochloride was coupled to **1**, to yield the corresponding mercapto-amide **5a**. Subsequent oxidation with peracetic acid afforded cholanic acid-taurine conjugate **5**.

*^a^* *Reagents and conditions:* (*i*) NMM, EDCI, CH_2_Cl_2_, r.t., overnight; (*ii*) NaOH (15% aq.), EtOH, r.t., 1 h.

*^a^* *Reagents and conditions:* (*i*) NMM, EDCI, CH_2_Cl_2_, r.t., overnight; (*ii*) NaOH (15% aq.), EtOH, r.t., 1 h.

*^a^* *Reagents and conditions:* (*i*) NMM, EDCI, CH_2_Cl_2_, r.t., overnight; (*ii*) CH_3_CO_2_H (40% aq.), CH_2_Cl_2_, 0 °C to r.t., 30 min.

### 2.2. Structure-Activity Relationship Analysis of Cholanic Acid Derivatives

Compounds **1**–**9** were evaluated for their ability to inhibit the binding of ephrin-A1 to the EphA2 receptor, by means of an ELISA assay protocol [14]. The pIC_50_ values of tested compounds are reported in Table 1, together with the corresponding standard deviations of the mean (SEM). Conjugation of cholanic acid with glycine provided compound **2** that was able to displace ephrin-A1 from the immobilized EphA2 receptor with an inhibitory potency (pIC_50_ = 4.88) comparable to that of cholanic acid (**1**). The methyl ester **3** resulted inactive, indicating that a free carboxylate group is fundamental to disrupt the interaction between the EphA2 receptor and the ephrin-A1 ligand. This result parallels EphA2-ephrin-A1 displacement data observed for the glycine and glycine methyl ester conjugates of LCA [16].

The β-alanine derivative **4** showed an inhibitory potency similar to **1** and **2** (pIC_50_ = 4.82), suggesting that the EphA2 receptor is tolerant to lengthening of the spacer between the terminal carboxyl group and the amide group. On the other hand, replacement of the carboxylic acid of compound **4** with a bioisosteric sulfonic acid (**5**) led to a loss of inhibitory activity, probably due to a detrimental arrangement of the sulfonate within the ligand-binding site of the receptor. Finally, conjugation with L-phenylalanine and L-tryptophan gave compounds **6** and **8**, that resulted less potent than **1** in the binding assay, with pIC_50_ values of 4.67 and 4.55, respectively. Finally, cholanic acid conjugates with D-phenylalanine (**7**) and D-tryptophan (**9**) had slightly lower pIC_50_ values than the corresponding L-amino acid derivatives **6** and **8**, highlighting a poor stereochemistry impact of the chiral amino acid portion.

In contrast with SAR for α-amino acid conjugates of LCA, where the introduction of an aromatic side chain (*i.e.* the 3-methylindole of tryptophan and the benzyl group of phenylalanine) resulted in a marked improvement of the inhibitory potency [16], in the case of cholanic acid derivatives, the same structural modification did not lead to an improvement of the EphA2-ephrin-A1 inhibitory potency, indicating that LCA conjugates and cholanic acid conjugates have a different SAR profile on the EphA2 receptor.

**Table 1:** pIC_5__0_ values for amino acid conjugates of cholanic acid tested on the EphA2 receptor.

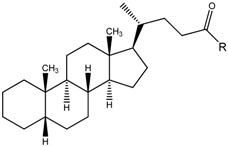		
Compound	R	pIC_50_±SEM *^a^*
**1**	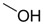	4.91 ± 0.09
**2**	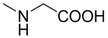	4.88 ± 0.06
**3**	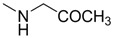	<3.50
**4**	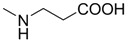	4.82 ± 0.07
**5**	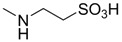	<3.50
**6**	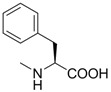	4.67 ± 0.07
**7**	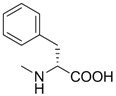	4.58 ± 0.07
**8**	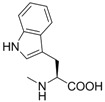	4.55 ± 0.08
**9**	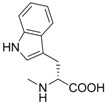	4.20 ± 0.11

*^a^* Values are means ± standard error of the mean (SEM) from at least three independent experiments.

### 2.3. Molecular Modeling Studies

**Figure 2:**
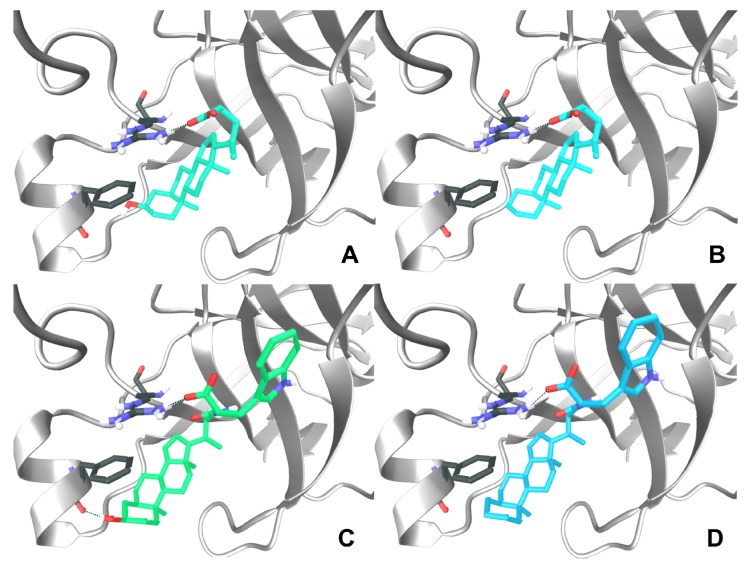
Lithocholic acid (**A**), cholanic acid (**B**), PCM 126 (**C**) and compound **8** (**D**) docked within the ligand binding domain of the EphA2 receptor. Secondary structure elements of EphA2 are colored in white, while carbon atoms of Arg103 and Phe156 are colored in grey (oxygens in red; nitrogens in blue, hydrogens in white). H-bond between the carboxylic acid of the ligands (**A**–**D**) and the guanidine group of Arg103 are highlighted with a dotted blue line. H-bond between the 3α-hydroxyl group of PCM126 and the backbone oxygen of Phe156 is depicted with a dotted green-line (**C**).

In our previous work, we proposed a binding mode for LCA to the EphA2 receptor based on docking and molecular dynamics simulations [15]. We speculated that this compound (LCA) might bind the EphA2 receptor by (*i*) inserting its cyclopenta[*a*]-perhydrophenanthrene scaffold into a large hydrophobic channel of the receptor and (*ii*) forming a key salt bridge with the guanidine group of Arg103, a critical residue involved in ephrin-A1 recognition [19]. In this model, the 3α-hydroxyl group, emerging from the A-ring of LCA, points towards the lipophilic side chain of Phe156 and does not undertake favorable interactions with the receptor (Figure 2A). This theoretical binding mode was supported by experimental data, as the removal of the hydroxyl group (LCA to cholanic acid, Figure 2B) led to a significant gain in potency [15].

Herein, we docked the L-Trp conjugate of both LCA (PCM126/UniPR126) and cholanic acid (compound **8**) within the EphA2 receptor to account for their experimental potency. The top-ranked binding poses (*i.e.*, those with the highest *Gscore*) are depicted in Figure 2C and Figure 2D. These poses are slightly different compared to those of the corresponding non-conjugated compounds. Indeed, the presence of the L-tryptophan moiety induced a shift of the whole structure to a lower position within the EphA2 channel both for PCM126 and compound **8**. In the case of PCM126, the small rearrangement of the binding mode allowed the formation of a hydrogen-bond between the 3α-hydroxyl group on the A-ring and the backbone oxygen of Phe156 (Figure 2C). This additional interaction could explain the higher inhibitory potency of PCM126 compared to LCA (pIC_50_ = 5.69 *vs.* pIC_50_ = 4.24). In the case of compound **8**, no additional hydrogen-bond can be formed, thus accounting for its reduced potency compared to PCM126 (pIC_50_ = 4.55 *vs.* pIC_50_ = 5.69). Further, the lipophilic A-ring of **8** undertakes an unfavorable interaction with the polar backbone oxygen of Phe156, accounting for its lower potency than free cholanic acid (pIC_50_ = 4.55 *vs.* pIC_50_ = 4.91).

### 2.4. Effects on EphA2 Phosphorylation in Human Prostate Adenocarcinoma Cells

**Figure 3:**
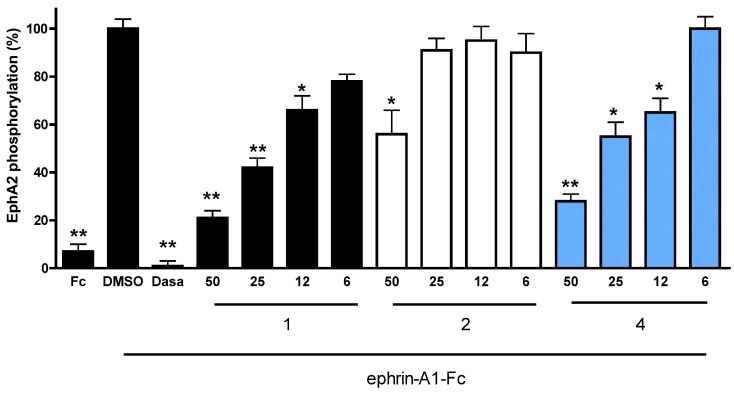
Relative EphA2 phosphorylation in the presence of different concentrations (50 μM, 25 μM, 12 μM, 6 μM) of compounds **1** (black), **2** (white) and **4** (sky blue). EphA2 phosphorylation was induced by treatment of PC3 cells with 0.25 μg/mL ephrin-A1-Fc. Cells were pretreated for 20 min with 1% DMSO or the indicated concentration of compounds and then stimulated for 20 min with ephrin-A1-Fc. Data are reported as a mean ± SEM of at least three independent experiments. One-way ANOVA followed by Dunnet’s post-test was performed to compare ephrin-A1-Fc + DMSO to all the other columns. * *p* < 0.05, ** *p* < 0.01.

Compounds **2** and **4**, which display an inhibitory potency comparable to that of cholanic acid in the ELISA binding assay, were evaluated in a functional study performed on PC3 human prostate adenocarcinoma cells that express the EphA2 receptor [20]. Cholanic acid (**1**) inhibited EphA2 phosphorylation induced by ephrin-A1-Fc, with an IC_50_ value of 17 µM. Similar to cholanic acid, **4** blocked EphA2 phosphorylation in a dose dependent manner with an IC_50_ of 21 µM, in agreement with the potency observed in the ELISA assay. Compound **2** was less potent than **1** in inhibiting EphA2 phosphorylation (Figure 3). The multikinase inhibitor dasatinib (1 µM), used as control, completely blocked EphA2 phosphorylation.

## 3. Experimental

### 3.1. Chemistry

#### General

Unless otherwise noted, reagents and solvents were purchased from commercial suppliers (Aldrich, Milano, Italy and Fluka, Milano, Italy) and were used without purification. The progress of the reaction was monitored by thin-layer chromatography with F_254_ silica-gel precoated sheets (Merck, Darmstadt, Germany). UV light, ninhydrin ethanolic solution (0.3% *w/v*) and potassium permanganate solution (10% *w/v*) were used for detection. Flash chromatography was performed using Merck silica-gel 60 (Si 60, 40–63 μm, 230–400 mesh ASTM). Dichloromethane was dried by distillation over calcium hydride. All reactions were carried out using flame-dried glassware under nitrogen atmosphere. Melting points were determined on a Gallenkamp melting point apparatus and were not corrected. The ^1^H-NMR (400 MHz) and ^13^C-NMR (100 MHz) spectra were recorded on a Bruker Avance 400 spectrometer; chemical shifts (*δ* scale) are reported in parts per million (ppm). ^1^H-NMR spectra are reported in the following order: multiplicity, approximate coupling constants (*J* value) in Hertz (Hz) and number of protons; signals were characterized as s (singlet), d (doublet), t (triplet), q (quadruplet), m (multiplet), b (broad). Mass spectra were recorded on an Applied Biosystems API-150 EX system spectrometer equipped with an ESI interface. The final compounds were analyzed on a ThermoQuest Italia (Milano, Italy). A FlashEA 1112 Elemental Analyzer was used for for C, H and N analyses. The percentages found were within ± 0.4% of the theoretical values.

*Methyl N-(5β-cholan-24-oyl)glycinate* (**3**). Compound **3** was synthesized following a modification of a described procedure [21] in which to a stirred solution of cholanic acid (**1**, 0.832 mmol), L-glycine methyl ester hydrochloride (0.915 mmol) and N-methylmorpholine (NMM, 2.080 mmol) in anhydrous CH_2_Cl_2_ (20 mL) under nitrogen was added *N*-(3-dimethylaminopropyl)-*N'*-ethylcarbodiimide hydrochloride (EDCI, 0.857 mmol). The reaction mixture was stirred at room temperature overnight and then was diluted with CH_2_Cl_2_, (30 mL) washed with HCl 2N, brine and dried over anhydrous Na_2_SO_4_. Evaporation of the solvent under reduced pressure yielded a white solid that was purified by flash chromatography [SiO_2_, CH_2_Cl_2_:EtOH 98:2]. The crude product was re-crystallized from ethanol-water to give **3**. Yield: 81%. Mp: 116–119 °C. ^1^H-NMR (CDCl_3_) *δ* = 0.63 (s, 3H, CH_3_), 0.90–0.92 (m, 7H), 1.00–1.13 (m, 4H), 1.14–1.30 (m, 8H), 1.32–1.42 (m, 7H), 1.53–1.57 (m, 1H), 1.71–1.74 (m, 3H), 1.78–1.87 (m, 3H), 1.92–1.95 (m, 1H), 2.09–2.16 (m, 1H), 2.26–2.33 (m, 1H), 3.75 (s, 3H, OCH_3_), 4.04 (d, *J* = 5.2 Hz, 2H, CH_2_CO), 6.02 (bs, 1H, NH). ^13^C-NMR (CDCl_3_) *δ* = 12.06, 18.37, 20.83, 21.34, 24.24, 24.26, 26.55, 27.03, 27.25, 27.51, 28.25, 31.61, 33.26, 35.36, 35.49, 35.89, 37.59, 40.29, 40.51, 41.20, 42.77, 43.73, 52.34, 56.05, 56.61, 170.64 (C=O), 173.78 (C=O). MS (ESI) calc for C_27_H_45_NO_3_: 431.34; found: 432.6 [M+H]^+^, 454.6 [M+Na]^+^, 470.5 [M+K]^+^. Anal. calc for C_27_H_45_NO_3_: C, 75.13; H, 10.51; N, 3.24; found: C, 75.77; H, 10.96; N, 3.11.

*N-(5β-cholan-24-oyl)glycine* (**2**). Compound **2** was synthesized following a modification of a described procedure [22] in which to a solution of compound **3** (0.563 mmol) in ethanol (15 mL) a solution of sodium hydroxide 15% *w/v* (12 mL) was added and the mixture stirred at room temperature for 1 h. Ethanol was removed under reduced pressure and the solution was acidified with concentrated hydrochloric acid until a precipitate was formed. The resulting suspension was suction-filtered and the obtained white residue washed with water. The crude product was crystallized from ethanol-water to give the title compound **2** (94%) as a white solid. Mp: 192–195 °C. ^1^H-NMR (DMSO-d_6_) *δ* = 0.60 (s, 3H, CH_3_), 0.81–0.88 (m, 7H), 0.94–1.09 (m, 4H), 1.13–1.25 (m, 9H), 1.32–1.34 (m, 6H), 1.49–1.51 (m, 1H), 1.65–1.84 (m, 6H), 1.90–1.92 (m, 1H), 1.97–2.04 (m, 1H), 2.09–2.16 (m, 1H), 3.69 (d, *J* = 6.0 Hz, 2H, CH_2_), 8.07 (t, *J* = 6.0 Hz, 1H, NH), 12.43 (bs, 1H, COOH). ^13^C-NMR (DMSO-d_6_) *δ* = 12.34, 18.75, 20.91, 21.28, 24.32, 24.50, 26.62, 26.96, 27.17, 27.51, 28.16, 31.90, 32.51, 35.34, 35.39, 35.86, 37.60, 40.99, 42.74, 43.57, 56.12, 56.53, 171.90 (C=O), 173.36 (C=O). MS (ESI) calc for C_26_H_43_NO_3_: 417.32; found: 416.6 [M−1]^−^. Anal. calc for C_26_H_43_NO_3_: C, 74.77; H, 10.38; N, 3.35; found: C, 74.88; H, 10.75; N, 3.32.

*Methyl N-(5β-cholan-24-oyl)-β-alaninate* (**4a**). Compound **4a** was synthesized following the procedure described for compound **3** using β-alanine methyl ester hydrochloride and purified by flash chromatography [SiO_2_, CH_2_Cl_2_:EtOH 98:2]. The crude product was re-crystallized from ethanol-water to give **4a**. Yield: 81%. Mp: 108–111 °C. ^1^H-NMR (CDCl_3_) *δ* = 0.63 (s, 3H, CH_3_), 0.90–092 (m, 7H), 1.01–1.14 (m, 4H), 1.16–1.32 (m, 8H), 1.34–1.41 (m, 6H), 1.54–1.57 (m, 1H), 1.61–1.65 (m, 1H), 1.72–1.88 (m, 6H), 1.92–1.96 (m, 1H), 2.00–2.08 (m, 1H), 2.18–2.25 (m, 1H), 2.54 (t, *J* = 6.0 Hz, 2H, CH_2_), 3.49–3.54 (m, 2H, CH_2_), 3.70 (s, 3H, OCH_3_), 6.02 (bs, 1H, NH). ^13^C-NMR (CDCl_3_) *δ* = 12.04, 18.36, 20.83, 21.34, 24.23, 24.27, 26.55, 27.03, 27.25, 27.51, 28.26, 31.74, 33.62, 33.86, 34.72, 35.36, 35.49, 35.89, 37.59, 40.29, 40.51, 42.76, 43.73, 51.78, 56.04, 56.61, 173.25 (C=O), 173.60 (C=O). MS (ESI) calc for C_28_H_47_NO_3_: 445.36; found: 446.5 [M+H]^+^, 468.5 [M+Na]^+^, 484.5 [M+K]^+^. Anal. calc for C_28_H_47_NO_3_: C, 75.46; H, 10.63; N, 3.14; found: C, 75.28; H, 10.56; N, 3.08.

*N-(5β-Cholan-24-oyl)-β-alanine* (**4**). Compound **4** was synthesized following a modification of a described procedure [23]. To a solution of compound **4a** (0.449 mmol) in ethanol (15 mL) a solution of sodium hydroxide 15% *w/v* (12 mL) was added and the mixture stirred at room temperature for 1 h. Ethanol was removed under reduced pressure and the solution was acidified with concentrated hydrochloric acid until a precipitate was formed. The resulting suspension was suction-filtered and the solid residue thus obtained washed with water. The crude product was re-crystallized from ethyl acetate to give the title compound **4**. Yield: 93%. Mp: 152–155 °C. ^1^H-NMR (DMSO-d_6_) *δ* = 0.59 (s, 3H, CH_3_), 0.84–0.88 (m, 7H), 1.03–1.08 (m, 4H), 1.15–1.34 (m, 15H), 1.50–1.58 (m, 1H), 1.65–1.71 (m, 4H), 1.76–1.84 (m, 2H), 1.89–1.96 (m, 2H), 2.01–2.08 (m, 1H), 2.32 (bt, *J* = 6.8 Hz, 2H, CH_2_), 3.16–3.21 (m, 2H, CH_2_), 7.82 (s, 1H, NH), 12.14 (bs, 1H, COOH). ^13^C-NMR (DMSO-d_6_) *δ* = 12.31, 18.74, 20.90, 21.28, 24.31, 24.50, 26.62, 26.96, 27.17, 27.51, 28.18, 32.00, 32.73, 34.42, 35.18, 35.39, 35.86, 37.60, 42.73, 43.57, 56.07, 56.53, 173.01 (C=O), 173.37 (C=O). MS (ESI) calc for C_27_H_45_NO_3_: 431.34; found: 430.6 [M−1]^−^. Anal. calc for C_27_H_45_NO_3_: C, 75.13; H, 10.51; N, 3.24; found: C, 75.20; H, 10.53; N, 3.18.

*2-[N-(5β-Cholan-24-oyl)amino]ethanethiol* (**5a**). Compound **5a** was synthesized following the procedure described for **3** using cysteamine hydrochloride and purified by flash chromatography [SiO_2_, CH_2_Cl_2_:MeOH(NH_3_) 98:2]. Yield: 36%. ^1^H-NMR (CDCl_3_) *δ* = 0.64 (s, 3H, CH_3_), 0.84–091 (m, 7H), 1.01–1.41 (m, 20H), 1.57 (m, 1H), 1.72–1.96 (m, 6H), 2.09–2.11 (m, 1H), 2.23–2.26 (m, 1H), 2.39 (m, 1H), 2.64–2.70 (m, 1H, CH_2_C*H*H), 3.41–3.46 (m, 1H, CH_2_CH*H*), 5.89 (bs, 1H, NH). ^13^C-NMR (CDCl_3_) *δ* = 12.08, 18.40, 20.83, 21.35, 24.26, 24.74, 26.56, 27.04, 27.25, 27.52, 28.29, 30.85, 31.81, 33.63, 35.36, 35.52, 35.90, 37.60, 40.30, 40.52, 42.28, 42.78, 43.74, 56.05, 56.62, 173.86 (C=O). MS (ESI) calc for C_26_H_45_NOS: 419.32; found: 420.5 [M+H]^+^, 442.4 [M+Na]^+^.

*2-[N-(5β-Cholan-24-oyl)amino]ethanesulfonic Acid* (**5**). Compound **5** was synthesized following a modification of a described procedure [24]. To a stirred solution of **5a** (0.295 mmol) in CH_2_Cl_2_ under nitrogen at 0 °C, a solution of peracetic acid in water (40% *w/w*, 0.966 mmol) was added dropwise The mixture was allowed to warm to room temperature while stirring over the course of 30 min. Evaporation of the solvent under reduced pressure afforded a white solid that was purified by flash chromatography [SiO_2_, CH_2_Cl_2_:HCOOH:EtOH 76:4:20]. The crude product was re-crystallized from ethanol-water to give **5**. Yield: 98%. Mp: 200–203 °C. ^1^H-NMR (CDCl_3_) *δ* = 0.59 (s, 3H, CH_3_), 0.84–0.88 (m, 7H), 1.01–1.08 (m, 5H), 1.14–1.22 (m, 9H), 1.31–1.34 (m, 6H), 1.51 (m, 1H), 1.61–1.71 (m, 4H), 1.76-1.81 (m, 2H), 1.84–1.94 (m, 2H), 2.00–2.07 (m, 1H), 7.65 (bs, 1H, NH). ^13^C-NMR (CDCl_3_) *δ* = 12.35, 18.77, 20.92, 21.30, 24.31, 24.50, 26.62, 26.99, 27.17, 27.53, 28.14, 31.93, 33.11, 35.37, 35.42, 35.91, 35.97, 37.64, 42.77, 43.62, 51.10, 56.09, 56.54, 172.51 (C=O). MS (ESI) calc for C_26_H_45_NO_4_S: 467.31; found: 466.4 [M−1]^−−^. Anal. calc for C_26_H_45_NO_4_S: C, 66.77; H, 9.70; N, 2.99; found: C, 66.60; H, 9.11; N, 2.68.

*Methyl N-(5β-cholan-24-oyl)-L-phenylalaninate* (**6a**). Compound **6a** was synthesized following procedure described for compound **3** starting from L-phenylalanine methyl ester hydrochloride and purified by flash chromatography [SiO_2_, CH_2_Cl_2_:EtOH 98:2]. The crude product was re-crystallized from ethanol-water to give **6a**. Yield: 83%. Mp: 78–81 °C. ^1^H-NMR (CDCl_3_) *δ* = 0.63 (s, 3H, CH_3_), 0.89–0.91 (m, 7H), 1.04–1.08 (m, 4H), 1.13–1.23 (m, 9H), 1.36–1.39 (m, 6H), 1.57 (m, 1H), 1.72–1.95 (m, 7H), 2.03–2.10 (m, 1H), 2.18–2.22 (m, 1H), 3.09 (dd, *J* = 13.8, 5.7 Hz, 1H, CHCH*H*), 3.15 (dd, *J* = 13.8, 5.8 Hz, 1H, CHC*H*H), 3.73 (s, 3H, OCH_3_), 4.87–4.92 (m, 1H, C*H*CH_2_), 5.86 (d, *J* = 7.7 Hz, 1H, NH), 7.09 (d, *J* = 6.4 Hz, 2H, Ar), 7.24-7.30 (m, 3H, Ar). ^13^C-NMR (CDCl_3_) *δ* = 12.07, 18.36, 20.83, 21.35, 24.25, 24.28, 26.56, 27.04, 27.26, 27.52, 28.24, 31.61, 33.40, 35.37, 35.44, 35.89, 37.60, 37.88, 40.29, 40.51, 42.76, 43.73, 52.32, 52.91, 56.01, 56.61, 127.12, 128.56, 129.28, 135.89, 172.21 (C=O), 173.15 (C=O). MS (ESI) calc for C_34_H_51_NO_3_: 521.39; found: 522.6 [M+H]^+^, 544.5 [M+Na]^+^. Anal. calc for C_34_H_51_NO_3_: C, 78.26; H, 9.85; N, 2.68; found: C, 78.62; H, 10.27; N, 2.67.

*N-(5β-Cholan-24-oyl)-L-phenylalanine* (**6**). Compound **6** was synthesized following the procedure described for compound **2** starting from compound **6a**. The crude product was re-crystallized from ethanol-water to give **5**. Yield: 97%. Mp: 189–192 °C. ^1^H-NMR (DMSO-d_6_) *δ* = 0.57 (s, 3H, CH_3_), 0.81–0.82 (m, 4H), 0.88 (s, 3H, CH_3_), 0.99–1.04 (m, 6H), 1.12–1.18 (m, 8H), 1.25–1.34 (m, 6H), 1.48–1.59 (m, 2H), 1.68–1.80 (m, 5H), 1.87–1.97 (m, 1H), 2.02–2.05 (m, 1H), 2.82 (dd, *J* = 13.6, 10.0 Hz, 1H, CHC*H*H), 3.02 (dd, *J* = 14.0, 4.8 Hz, 1H, CHCH*H*), 4.34–4.40 (m, 1H, C*H*CH_2_), 7.15–7.26 (m, 5H, Ar), 8.08 (d, *J* = 8.4 Hz, 1H, NH). ^13^C-NMR (DMSO-d_6_) *δ* = 12.32, 18.72, 20.90, 21.28, 24.31, 24.51, 26.62, 26.96, 27.17, 27.51, 28.11, 31.90, 32.50, 35.30, 35.39, 35.86, 37.20, 37.60, 42.70, 43.57, 53.78, 56.05, 56.52, 126.76, 128.53, 129.52, 138.26, 173.03 (C=O), 173.66 (C=O). MS (ESI) calc for C_33_H_49_NO_3_: 507.37; found: 506.6 [M−H]^−−^. Anal. calc for C_33_H_49_NO_3_: C, 78.06; H, 9.73; N, 2.76; found: C, 78.34; H, 9.40; N, 2.76.

*Methyl N-(5β-cholan-24-oyl)-D-phenylalaninate* (**7a**). Compound **7a** was synthesized following the procedure described for compound **3** starting from D-phenylalanine methyl ester hydrochloride and purified by flash chromatography [SiO_2_, CH_2_Cl_2_:C_2_H_5_OH 99:1]. The crude product was re-crystallized from ethanol-water to give **7a**. Yield: 90%. Mp: 82–85 °C. ^1^H-NMR (CDCl_3_) *δ* = 0.63 (s, 3H, CH_3_), 0.88–0.91 (m, 7H), 1.04–1.13 (m, 5H), 1.16–1.23 (m, 8H), 1.36–1.38 (m, 6H), 1.57 (m, 1H), 1.65 (m, 1H), 1.72–1.85 (m, 6H), 1.93–1.96 (m, 1H), 2.05–2.09 (m, 1H), 2.20–2.24 (m, 1H), 3.09 (dd, *J* = 13.84, 5.68 Hz, 1H, CHCH*H*), 3.15 (dd, *J* = 13.84, 5.84 Hz, 1H, CHC*H*H ), 3.73 (s, 3H, OCH_3_), 4.87–4.92 (m, 1H, C*H*CH_2_), 5.86 (d, *J* = 7.6 Hz, 1H, NH), 7.08–7.09 (m, 2H, Ar), 7.24–7.30 (m, 3H, Ar). ^13^C-NMR (CDCl_3_) *δ* = 12.09, 18.33, 20.84, 21.35, 24.24, 24.28, 26.56, 27.04, 27.26, 27.53, 28.24, 31.60, 33.47, 35.37, 35.48, 35.90, 37.60, 37.91, 40.30, 40.52, 42.77, 43.74, 52.30, 52.90, 56.07, 56.61, 127.11, 128.55, 129.28, 135.89, 172.18 (C=O), 173.05 (C=O). MS (ESI) calc for C_34_H_51_NO_3_: 521.39; found: 522.5 [M+H]^+^, 544.3 [M+Na]^+^. Anal. calc for C_34_H_51_NO_4_: C, 78.26; H, 9.85; N, 2.68; found: C, 78.62; H, 10.27; N, 2.67.

*N-(5β-cholan-24-oyl)-D-phenylalanine* (**7**). Compound **7** was synthesized following the procedure described for **2** using **7a**. The crude product was re-crystallized from ethanol-water to give **7**. Yield: 97%. Mp: 195–198 °C. ^1^H-NMR (DMSO-d_6_) *δ* = 0.56 (s, 3H, CH_3_), 0.79–0.81 (m, 3H), 0.83–0.88 (m, 4H), 0.99-1.04 (m, 5H), 1.15–1.18 (m, 9H), 1.31–1.32 (m, 5H), 1.50–1.55 (m, 2H), 1.68–1.96 (m, 7H), 2.04–2.07 (m, 1H), 2.82 (dd, *J* = 13.52, 8.96 Hz, 1H, CHC*H*H), 3.03 (dd, *J* = 13.64, 4.68 Hz, 1H, CHCH*H*), 4.31–4.32 (m, 1H, C*H*CH_2_), 7.14–7.22 (m, 5H, Ar), 7.86 (d, *J* = 7.64 Hz, 1H, NH). ^13^C-NMR (CDCl_3_) *δ* = 12.33, 18.64, 20.91, 21.28, 24.31, 24.51, 26.62, 26.95, 27.17, 27.51, 28.14, 31.92, 32.75, 35.30, 35.38, 35.84, 37.51, 37.60, 42.70, 43.56, 54.46, 56.22, 56.50, 126.49, 128.34, 129.65, 138.78, 172.52 (C=O), 173.96 (C=O). MS (ESI) calc for C_33_H_49_NO_3_: 507.37; found: 506.5 [M−H]^−−^. Anal. calc for C_33_H_49_NO_4_: C, 78.06; H, 9.73; N, 2.76; found: C, 78.34; H, 9.40; N, 2.76.

*Methyl N-(5β-cholan-24-oyl)-L-tryptophanate* (**8a**). Compound **8a** was synthesized following the procedure described for compound **3** starting from L-tryptophan methyl ester hydrochloride and purified by flash chromatography [SiO_2_, CH_2_Cl_2_:EtOH 98:2], yielding compound **8a** as a pale-yellow oil, which was used in the next step without further purification. Yield: 98%. ^1^H-NMR (DMSO-d_6_) *δ* = 0.62 (s, 3H, CH_3_), 0.83–0.91 (m, 7H), 0.99–1.09 (m, 5H), 1.12–1.30 (m, 9H), 1.35–1.38 (m, 6H), 1.55–1.57 (m, 1H), 1.73–2.09 (m, 7H), 2.18–2.26 (m, 1H), 3.32 (dd, *J* = 5.2, 2.1 Hz, 2H, CHC*H*_2_), 3.70 (s, 3H, OCH_3_), 4.96 (dt, *J* = 7.8, 5.4 Hz, 1H, C*H*CH_2_), 6.00 (bd, *J* = 8.1 Hz, 1H, NH), 6.97 (d, *J* = 2.4 Hz, 1H, Ar), 7.12 (td, *J* = 7.2, 1.2 Hz 1H, Ar), 7.19 (td, *J* = 7.2, 1.2 Hz 1H, Ar), 7.36 (d, *J* = 8.1 Hz, 1H, Ar), 7.53 (d, *J* = 8.1 Hz, 1H, Ar), 8.32 (bs, 1H, NH). ^13^C-NMR (DMSO-d_6_) *δ* = 12.05, 18.35, 20.83, 21.35, 24.24, 24.28, 26.57, 27.04, 27.26, 27.53, 27.65, 28.22, 31.51, 33.45, 35.37, 35.43, 35.89, 37.60, 40.28, 40.52, 42.75, 43.74, 52.33, 52.93, 56.00, 56.60, 110.17, 111.28, 118.59, 119.72, 122.25, 122.67, 127.76, 136.11, 172.33 (C=O), 173.33 (C=O). MS (ESI) calc for C_36_H_52_N_2_O_3_: 560.40; found: 561.7 [M+H]^+^, 583.6 [M+Na]^+^, 599.5 [M+K]^+^.

*N-(5β-cholan-24-oyl)-L-tryptophan* (**8**). Compound **8** was synthesized following the procedure described for **2** using **8a**. The crude product was re-crystallized from ethanol-water to give **8**. Yield: 80%. Mp: 98–101 °C. ^1^H-NMR (DMSO-d_6_) *δ* = 0.57 (s, 3H, CH_3_), 0.80–0.88 (m, 7H), 0.97–1.37 (m, 19H), 1.48–1.57 (m, 2H), 1.68–1.92 (m, 7H), 1.98–2.06 (m, 1H), 2.97 (dd, *J* = 14.4, 5.6 Hz, 1H, CHC*H*H), 3.19 (dd, *J* = 14.4, 5.2 Hz, 1H, CHCH*H*), 4.12 (q, *J* = 5.6 Hz, 1H, C*H*CH_2_), 6.87 (t, *J* = 7.6 Hz, 1H, Ar), 6.97 (t, *J* = 7.2 Hz, 1H, Ar), 7.02 (d, *J* = 1.6 Hz, 1H,, Ar), 7.17 (d, *J* = 7.2 Hz, 1H, NH), 7.26 (d, *J* = 8.0 Hz, 1H, Ar), 7.46 (d, *J* = 8.0 Hz, 1H, Ar). ^13^C-NMR (DMSO-d_6_) *δ* = 12.33, 18.77, 20.90, 21.28, 24.33, 24.51, 26.62, 26.96, 27.17, 27.52, 28.05, 28.15, 31.91, 33.19, 35.40, 35.46, 35.86, 37.61, 42.70, 43.58, 55.45, 56.07, 56.48, 111.42, 112.13, 118.17, 119.06, 120.72, 123.64, 128.74, 136.32, 171.70 (C=O), 174.85 (C=O). MS (ESI) calc for C_35_H_50_N_2_O_3_: 546.38; found: 545.7 [M−H]^−^. Anal. calc for C_35_H_50_N_2_O_3_ • 0.2 H_2_O: C, 76.37; H, 9.23; N, 5.09; found: C, 76.07; H, 9.61; N, 4.78.

*d-Tryptophan methyl ester hydrochloride* (**9b**)*.* Compound **9b** was synthesized following a described procedure [25] starting from d-tryptophan.

*Methyl N-(5β-cholan-24-oyl)-D-tryptophanate* (**9a**). Compound **9a** was synthesized following the procedure described for compound **3** starting from D-tryptophan methyl ester hydrochloride **9b** and purified by flash chromatography [SiO_2_, CH_2_Cl_2_:C_2_H_5_OH 98:2], to furnish compound **9a** as a pale-yellow oil. Yield: 81%. ^1^H-NMR (DMSO-d_6_) *δ* = 0.61 (s, 3H, CH_3_), 0.85–0.91 (m, 7H), 1.02–1.38 (m, 20H), 1.53–1.58 (m, 1H), 1.72–2.04 (m, 7H), 2.15–2.27 (m, 1H), 3.31 (d, *J* = 5.3 Hz, 2H, CHC*H*_2_), 3.68 (s, 3H, OCH_3_), 4.96 (dt, *J* = 7.8, 5.3 Hz, 1H, C*H*CH_2_), 6.95 (d, *J* = 1.9 Hz, 1H, Ar), 7.10 (td, *J* = 6.9, 1.2 Hz 1H, Ar), 7.18 (td, *J* = 6.9, 1.2 Hz 1H, Ar), 7.34 (d, *J* = 7.9 Hz, 1H, Ar), 7.52 (d, *J* = 7.7 Hz, 1H, Ar), 8.43 (bs, 1H, NH). ^13^C-NMR (DMSO-d_6_) *δ* = 12.05, 18.40, 20.90, 21.42, 24.30, 24.35, 26.63, 27.11, 27.33, 27.60, 27.74, 28.27, 31.57, 33.50, 35.43, 35.52, 35.96, 37.67, 40.35, 40.58, 42.82, 43.81, 52.39, 53.02, 56.06, 56.65, 110.06, 111.41, 118.59, 119.72, 122.25, 122.83, 127.80, 136.23, 172.63 (C=O), 173.44 (C=O). MS (ESI) calc for C_36_H_52_N_2_O_3_: 560.40; found: 561.6 [M+H]^+^, 583.4 [M+Na]^+^, 599.5 [M+K]^+^.

*N-(5β-cholan-24-oyl)-D-tryptophan* (**9**). Compound **9** was synthesized following the procedure described for **2** using compound **9a**. The crude product was re-crystallized from ethanol-water to give **9**. Yield: 77%. Mp: 141–145 °C. ^1^H-NMR (DMSO-d_6_) *δ* = 0.56 (s, 3H, CH_3_), 0.82–0.88 (m, 7H), 1.00–1.34 (m, 19H), 1.47–1.62 (m, 2H), 1.69–1.98 (m, 7H), 2.05–2.09 (m, 1H), 2.97 (dd, *J* = 14.4, 8.4 Hz, 1H, CHC*H*H), 3.15 (dd, *J* = 14.4, 4.6 Hz, 1H, CHCH*H*), 4.38–4.43 (m, 1H, C*H*CH_2_), 6.95 (t, *J* = 7.2 Hz, 1H, Ar), 7.04 (t, *J* = 7.4 Hz, 1H, Ar), 7.10 (s, 1H, Ar), 7.31 (d, *J* = 8.0 Hz, 1H, Ar), 7.51 (d, *J* = 7.6 Hz, 1H, Ar), 7.94 (bd, *J* = 7.6 Hz, 1H, NH), 10.8 (s, 1H, COOH). ^13^C-NMR (DMSO-d_6_) *δ* = 11.81, 18.23, 20.42, 20.81, 23.84, 24.05, 26.15, 26.48, 26.69, 27.04, 27.18, 27.64, 31.37, 32.17, 34.84, 34.93, 35.39, 37.13, 42.23, 43.09, 53.18, 55.65, 56.03, 110.25, 111.27, 118.21, 120.77, 123.41, 127.36, 136.04, 172.36, 173.80. MS (ESI) calc for C_35_H_50_N_2_O_3_: 546.38; found: 545.5 [M−H]^−^. Anal. calc for C_35_H_50_N_2_O_3_ • 0.5 H_2_O: C, 75.63; H, 9.25; N, 5.04; found: C, 75.58; H, 9.46; N, 4.58.

### 3.2. Molecular Modeling

The crystal structure of the EphA2–ephrin-A1 complex (PDB accession 3HEI) [19] was used for molecular modeling simulations. Chains A and B of the crystal structure were extracted and processed with the Protein Preparation Wizard tool implemented in Maestro [26], which was used to assign bond orders and to add missing hydrogen atoms. The overall hydrogen bonding network was optimized by sampling the sidechain amide-groups orientation of asparagines and glutamines, the hydroxyl-group of serine and threonine residues and the thiol-group of cysteine residues and water molecules, and finally by adjusting the tautomeric forms of histidines. A final restrained minimization was conducted with the OPLS2005 force field to a root mean square deviation value of 0.3 Å calculated on protein heavy atoms. Small molecules were built with Maestro 9.2 and energy-minimized applying the OPSL2005 force field and the GBSA water solvation treatment to a gradient of 0.05 kj•mol-1•Å^−1^. The minimized ligand structures were then docked within the EphA2 receptor using Glide5.5 [27] in standard precision mode and applying a hydrogen bond constraint between the carboxylic group of the compound and the guanidine group of Arg103. Docking grids were centered within the EphA2 receptor binding site in a region delimited by Arg103, Phe156 and Arg159; the dimensions of enclosing and bounding boxes were set to 29.4 and 10 Å, respectively. Van der Waals (VdW) radii of protein atoms were not scaled, while a scaling factor of 0.8 was applied to the VdW radii of ligand atoms having partial atomic charges lower than |0.15|. Twenty docking poses were retained for each ligand and ranked according to the *Gscore*. Log P calculation (AlogP) for cholanic acid and compound **4** were performed with Canvas [28].

### 3.3. Pharmacology

#### 3.3.1. Reagents

All culture media and supplements were purchased from Euroclone (Milano, Italy). Recombinant proteins and antibodies were from R&D Systems (Minneapolis, MN, USA). Cells were purchased from ECACC (Salisbury, UK). Leupeptin, aprotinin, NP40, tween20, BSA and salts for solutions were from Applichem (Darmstadt,Germany); EDTA and sodium orthovanadate were from Sigma (Milano, Italy). Human IgG Fc fragment was from Millipore (AG714, Darmstadt, Germany).

#### 3.3.2. Cell Cultures

PC3 human prostate adenocarcinoma cells were grown in Ham F12 media and supplemented with 5% fetal bovine serum (FBS) and 1% antibiotic solution. PC3 cells were grown in a humidified atmosphere of 95% air, 5% CO_2_ at 37 °C.

#### 3.3.3. ELISA Assays and IC_50_ Determination

ELISA assays were performed as previously described [14]. Briefly, compounds were stocked as 5 mM solutions in dimethyl sulfoxide (DMSO) and tested in displacing studies, starting from a concentration of 50 µM. Ninety-six well ELISA high binding plates (2592, Costar, Amsterdam, The Netherlands) were incubated overnight at 4 °C with 100 µL/well of 1 µg/mL EphA2-Fc (R&D 639-A2) diluted in sterile phosphate buffered saline (PBS, 0.2 g/L KCl, 8.0 g/L NaCl, 0.2 g/L KH_2_PO_4_, 1.15 g/L Na_2_HPO_4_, pH 7.4). The wells were then washed with washing buffer (PBS +0.05% tween20, pH 7.5) and treated with blocking solution (PBS + 0.5% BSA) for 1 h at 37 °C. Compounds were added to the wells at proper concentration in 1% DMSO and incubated at 37 °C for 1 h. Biotinylated ephrin-A1-Fc (R&D Systems BT602) was added at 37 °C for 4 h at its K_D_ in displacement assays. The wells were washed and incubated with 100 µL/well streptavidin-HRP (Sigma S5512) in blocking solution (0.05 µg/mL in PBS supplemented with 0.5% BSA, pH 7.4) for 20 min at room temperature, then washed again and incubated at room temperature with 0.1 mg/mL tetramethylbenzidine (Sigma T2885) reconstituted in stable peroxide buffer (11.3 g/L citric acid, 9.7 g/L sodium phosphate, pH 5.0) and 0.02% H_2_O_2_ (30% m/m in water), added immediately before use. The reaction was stopped with 3N HCl 100 µL/well and the absorbance was measured using an ELISA plate reader (Sunrise, TECAN, Männedorf, Switzerland) at 450 nm. IC_50_ values were determined using one-site competition non-linear regression analysis with Prism software (GraphPad Software Inc., San Diego, CA, USA).

#### 3.3.4. Phosphorylation of EphA2 in Cells

PC3 cells were seeded in 12-well plates at concentration of 10^5^ cells/mL, 1 mL/well, in complete medium until they reached ~70% confluence and serum starved overnight. Thereafter, the cells were treated with the compounds under study, vehicle or standard drug, stimulated with ephrin-A1-Fc, rinsed with sterile PBS and solubilized in lysis buffer (1% NP-40, 20 mM Tris (pH 8.0), 137 mM NaCl, 10% glycerol, 2 mM EDTA, 1 mM activated sodium orthovanadate, 10 µg/mL Aprotinin, 10 µg/mL Leupeptin). The lysates were resuspended and rocked at 4 °C for 30 min and then centrifuged at 14,000g for 5 min. The protein content of supernatant was measured with BCA protein assay kit (Thermo Fisher scientific, Pittsburgh, PA) and standardized to 200 µg/mL. EphA2 phosphorylation was measured in cell lysates using a DuoSet®IC Sandwich ELISA (R&D Systems, DYC4056) following the manufacturer’s protocol: 96 well ELISA high binding plates (Costar 2592) were incubated overnight at room temperature with 100 µL/well of EphA2 capture antibody diluted in sterile PBS to the proper working concentration. After blocking, the wells were incubated for 2 h at room temperature with 100 µL/well of lysates, followed by incubation with the detection antibody at room temperature for 2 h. Receptor phosphorylation was revealed utilizing a standard HRP format with a colorimetric reaction read at 450 nm.

## 4. Conclusions

The pivotal role of the Eph-ephrin system in tumor growth and angiogenesis makes increasingly necessary the development of pharmacological tools to better clarify the cellular functions regulated by this system, as well as to investigate the potential antitumor action resulting from the blockage of the Eph-ephrin signaling. In this scenario, bile acids such as lithocholic acid and cholanic acid [14,15] enter as promising EphA2 antagonists being able to disrupt the EphA2-ephrin-A1 complex in binding assay and to inhibit EphA2 activation in cells.

Prompted by the observation that conjugation of lithocholic acid with naturally occurring amino acids yielded potent and effective EphA2 antagonists [16], in the present work we prepared a set of cholanic acid derivatives obtained by conjugation with a panel of amino acids with the aim of improving the inhibitory potency on the EphA2 receptor.

With the exception of the methyl ester **3** and the sulfonic acid derivative **5**, all the synthetized cholanic acid conjugates were indeed able to disrupt the EphA2-ephrin-A1 interaction, suggesting that, after conjugation, the bioisostery between lithocholic and cholanic acid is maintained at some extent. 

However, in contrast to what observed for LCA conjugates, no improvement was observed, when cholanic acid was conjugated to the aromatic L-tryptophan and L-phenylalanine. Moreover, the pair comparison between the LCA derivative PCM126 and the cholanic derivative **8** indicated that the 3α-hydroxyl group is vital to obtain potent EphA2 binders. On the other hands, our exploration identified the β-alanine derivative **4** (AlogP = 5.0) as an effective antagonist of EphA2 receptor, featuring a slightly lower lipophilicity than cholanic acid (Alog P = 5.8) and thus more promising for a future chemical expansion. These findings will help the design and synthesis of novel antagonists of the Eph-ephrin system.
